# Treatment decision-making for leptomeningeal metastasis after TKI resistance in EGFR-mutant lung cancer: a comprehensive review on staging, resistance patterns, and multimodal interventions

**DOI:** 10.3389/fneur.2026.1737679

**Published:** 2026-05-21

**Authors:** Guo-Qiang Song, Tian-li He, Ke-jie Ji, Yi-meng Duan, Guo-qiang Hu

**Affiliations:** 1 Department of Respiratory Medicine, Changxing County Hospital of Traditional Chinese Medicine, Changxing County, Zhejiang, China; 2 Department of Radiotherapy, Changxing County People's Hospital, Changxing County, Zhejiang, China

**Keywords:** EGFR mutation, intracranial pressure relief device, intrathecal injection, leptomeningeal metastasis, non-small cell lung cancer, radiotherapy, TKI resistance, treatment decision-making

## Abstract

Leptomeningeal metastasis (LM) represents a devastating complication in patients with EGFR-mutant non-small cell lung cancer (NSCLC) who develop resistance to tyrosine kinase inhibitors (TKIs). The management of LM after TKI resistance poses significant clinical challenges due to the heterogeneity of tumor staging, diverse resistance mechanisms, and limited penetration of systemic therapies across the blood–brain barrier (BBB). Current treatment strategies lack standardized protocols and require careful consideration of tumor burden, resistance patterns, and patient-specific factors. This narrative review systematically summarizes recent clinical studies, guidelines, and expert consensus addressing therapeutic decision-making in this setting. This review emphasizes multimodal clinical decision-making, integrating targeted therapies, intrathecal drug administration, radiotherapy, and intracranial pressure relief devices, with discussion of emerging immunological strategies. Although combining these therapies can improve neurological prognosis and survival rates for some patients, toxicity, the unpredictability of efficacy, and the logistical challenges of multidisciplinary treatment remain significant obstacles. To address this issue, we outline a clinical decision-making pathway aimed at helping physicians optimize and personalize treatment regimens. Looking ahead, improving our methods for identifying drug resistance mechanisms, optimizing central nervous system (CNS) drug delivery, and building stronger collaborative care models will be crucial for improving the prognosis of this vulnerable group.

## 1 Introduction

Epidermal growth factor receptor (EGFR) mutations represent the most common molecular subtype of non-small cell lung cancer (NSCLC) in Asian populations. While first- and second-generation EGFR tyrosine kinase inhibitors (TKIs) markedly improve progression-free survival (PFS) and quality of life for patients with sensitizing mutations (e.g., exon 19 deletions or L858R), third-generation agents like Osimertinib have pushed clinical outcomes further. By targeting T790M resistance mutations and offering robust central nervous system (CNS) penetration, these newer TKIs directly address the high risk of brain metastases (1–3). Despite these advances, acquired resistance to EGFR-TKIs remains inevitable and represents a formidable obstacle in the management of EGFR-mutant NSCLC. The molecular mechanisms underlying resistance are multifaceted, including secondary EGFR mutations (e.g., C797S), activation of bypass signaling pathways such as MET amplification, HER3 upregulation, and alterations in tumor microenvironment components, including tumor-associated macrophages and exosomal non-coding RNAs (4–7). Elucidating these resistance mechanisms has driven the development of new combination therapies. By integrating next-generation TKIs with antiangiogenic drugs or immune checkpoint inhibitors, current strategies aim to b2ypass resistance and extend patient survival (8–10).

Following TKI resistance in EGFR-mutant NSCLC, leptomeningeal metastasis (LM)—the spread of tumor cells into the cerebrospinal fluid (CSF) and leptomeninges—has emerged as a critical complication. Paradoxically, the incidence of LM is rising; as better systemic treatments prolong patient survival, CNS metastases have more time to develop. Once LM occurs, the prognosis remains exceptionally poor, with median overall survival (OS) typically limited to a few months even with aggressive intervention (11, 12). Treating LM remains notoriously difficult because intact anatomical boundaries—specifically the blood–brain (BBB) and blood-CSF barriers—severely restrict drug delivery to the CNS. Moreover, the diagnosis of LM is complicated by heterogeneous clinical presentations and the limited sensitivity of CSF cytology. Recent advances in liquid biopsy techniques analyzing CSF ctDNA have improved the detection of driver mutations and resistance mechanisms within the CNS compartment, enabling more precise therapeutic decision-making (13–15).

Once TKI resistance and LM develop in EGFR-mutant NSCLC, clinical decision-making becomes highly complex. Treatment strategies must be meticulously tailored to individual patient conditions, considering factors such as performance status, systemic and intracranial disease burden, and potential molecular resistance mechanisms. For patients harboring the T790M mutation after first- or second-generation TKI failure, osimertinib (standard 80 mg or dose-escalated 160 mg daily) remains the preferred third-generation agent per AURA3 and NCCN Category 1 recommendations; high-dose regimens are particularly relevant in LM owing to improved CSF exposure (16–18). For those with other driver factors (e.g., MET amplification or HER3 upregulation), combination therapy is typically required to effectively block these pathways (19, 20). Per the latest NCCN NSCLC Guidelines (Version 3.2025), Category 1 recommendations for newly diagnosed EGFR exon 19del/L858R disease include Osimertinib monotherapy (21), Osimertinib plus platinum-pemetrexed (22, 23), or Amivantamab plus Lazertinib (24). These regimens have substantially delayed CNS progression, yet acquired resistance and LM remain inevitable in a subset of patients. Furthermore, the use of local therapies such as stereotactic radiosurgery (SRS), whole-brain radiotherapy (WBRT), and intrathecal chemotherapy can be integrated into multimodal strategies to enhance intracranial disease control. Studies have demonstrated that combining local ablative radiotherapy with EGFR-TKIs can delay progression and improve survival outcomes in selected patients (25, 26). Despite these advances, the optimal approach to prioritizing and combining systemic and local treatments remains unclear. The lack of randomized controlled trials, coupled with the complex interplay between tumor biology and central nervous system pharmacokinetics, makes it difficult to establish definitive standard treatment protocols.

Given the absence of standardized treatment guidelines for LM after TKI resistance, a multidisciplinary approach incorporating molecular profiling via CSF liquid biopsy, radiological assessment, and evaluation of patient-specific factors is essential. Emerging evidence supports the trend toward multimodal interventions, including intrathecal administration of chemotherapeutic agents like pemetrexed, high-dose third-generation EGFR-TKIs such as Osimertinib or Furmonertinib, and adjunctive radiotherapy, tailored to the resistance pattern and disease extent (27–29). Additionally, novel agents targeting resistance mechanisms and immune microenvironment modulation, including CTLA4 blockade, are under investigation and may offer future therapeutic avenues (30, 31).

In this comprehensive review, we analyze treatment options for EGFR-mutant NSCLC patients with LM following TKI resistance, stratified by disease stage and resistance mechanisms. We explore the role of molecular diagnostics, including CSF ctDNA analysis, in guiding therapy selection. In addition, we examined the clinical evidence supporting multimodal interventions such as intrathecal chemotherapy, cranial irradiation, and intracranial pressure management (including ventriculoperitoneal shunting). Finally, we proposed a clinical decision algorithm integrating molecular, imaging, and clinical parameters to optimize individualized treatment strategies for this challenging patient population.

## 2 Methods

### 2.1 Literature search methodology

This manuscript is a narrative review synthesizing current evidence on the management of LM in patients with EGFR-mutant NSCLC following TKI resistance. A comprehensive literature search was conducted in the PubMed, Embase, and Google Scholar databases for articles published between January 2015 and December 2025. Search terms included combinations of ‘EGFR-mutant NSCLC,’ ‘leptomeningeal metastasis,’ ‘TKI resistance,’ and ‘CNS outcomes.’ Inclusion criteria focused on English-language, human studies evaluating CNS outcomes, including randomized controlled trials (RCTs), prospective and retrospective cohorts, case reports, and clinical guidelines. Evidence was prioritized based on its clinical strength: prospective data and established clinical guidelines (e.g., National Comprehensive Cancer Network (NCCN), European Association of Neuro-Oncology-European Society for Medical Oncology (EANO-ESMO)) were given precedence over retrospective series and individual case reports to minimize selection bias and ensure the reliability of the proposed clinical decision-making roadmap.

### 2.2 Established a grading standard

We defined four-tier evidence grading system. Recommendations are graded as (A) guideline/consensus-supported (e.g., EANO-ESMO), (B) prospective trials/phase II, (C) retrospective cohorts, and (D) case reports/experimental/preclinical.

## 3 Epidemiology and clinical characteristics of leptomeningeal metastasis after TKI resistance in EGFR-mutant lung cancer

### 3.1 Incidence, risk factors, and pathophysiology of leptomeningeal metastasis

LM represents a devastating complication in patients with EGFR-mutant NSCLC, with significant clinical implications for prognosis and management. The incidence of LM in EGFR-mutant NSCLC ranges from 9.8 to 13.7% (32, 33). These data underscore the non-negligible risk of LM in EGFR-mutated NSCLC, especially in the context of pre-existing intracranial disease. While this review focuses on EGFR-mutant NSCLC, primarily adenocarcinoma (where EGFR mutations predominate in 40–50% of Asian cases), LM can also occur in other lung cancer subtypes. For instance, small cell lung cancer (SCLC) accounts for 10–15% of LM cases overall, often due to its aggressive nature, though EGFR mutations are rare in SCLC. Squamous cell NSCLC and large cell carcinoma show lower LM incidence, highlighting the role of molecular drivers like EGFR in CNS tropism (34, 35).

The risk of LM is not uniformly distributed, and certain clinical and molecular factors significantly increase susceptibility. EGFR mutation itself has been consistently identified as an independent risk factor for LM development. For instance, in a cohort of 784 patients with brain metastases, mutated EGFR was associated with a higher likelihood of developing type I LM both at brain metastases (BM) onset and during follow-up (P: 0.004 and *p* < 0.0001, respectively) (36). Furthermore, younger age and poor performance status were found to correlate with increased LM risk. In another study, younger patients (hazard ratio [HR] 1.043 per year decrease, *p* < 0.001), initial presentation as metastatic disease (HR 3.768, p < 0.001), and presence of brain or lung metastases at diagnosis (HR 8.682 and HR 2.317, respectively) were all linked to higher LM incidence (32). These findings suggest that aggressive disease biology and early dissemination may predispose certain subgroups to LM.

The dissemination of tumor cells to the leptomeninges occurs via multiple pathways, with hematogenous spread being the predominant mechanism. Cancer cells primarily reach the CSF through the choroid plexus or arachnoid vessels, often via fenestrated capillaries or Batson’s venous plexus, allowing entry without crossing the blood–brain barrier directly. Local invasion from adjacent brain parenchyma, dura, or cranial nerves is less common but seen in advanced intracranial disease. Lymphatic involvement plays a minor role, typically via perineural or centripetal extension from axial lymph nodes or along emissary veins connecting bone marrow to the meninges; however, lymphatic vessel invasion (LVI) may facilitate early seeding in some cases, influenced by factors like integrin α6 expression on tumor cells. In EGFR-mutant NSCLC, epithelial-mesenchymal transition (EMT) and VEGF secretion enhance vascular permeability and CNS tropism (34, 37, 38).

Risk stratification may also largely depend on potential EGFR molecular subtypes. Most literature emphasizes common mutations, but emerging data suggests that rare mutations, such as exon 20 insertions, have unique and potentially more invasive clinical course. This was reflected in a recent case report that tracked a patient with exon 20 insertion who experienced rapid progression of meningeal metastasis only 11 months after radical surgery (39). This observation raises the possibility that specific EGFR mutation subtypes could confer differential propensities for CNS dissemination and therapeutic resistance, although larger studies are needed to confirm this trend.

Clinical history also plays a pivotal role in determining LM risk. The presence of brain metastases at baseline is a particularly strong predictor; in one analysis, patients with prior brain metastasis had an almost ninefold increased risk of subsequent LM compared to those without (32). Additionally, tumor burden—specifically, larger initial intracranial tumor volume and the presence of more than five brain lesions—was independently associated with post-SRS LM development (33). These data highlight the importance of careful surveillance and possibly more aggressive CNS-directed therapies in patients with extensive intracranial disease at presentation.

Therapeutic factors also modulate LM risk. Use of third-generation EGFR-TKIs such as Osimertinib has been shown to significantly reduce the incidence of LM in EGFR-mutated NSCLC. In a propensity-matched retrospective study, the cumulative incidence of LM was 9.82% in patients treated with Osimertinib versus 21.42% in those who did not receive Osimertinib, with multivariate analysis confirming Osimertinib as an independent protective factor (HR 0.33, P: 0.042) (40). This protective effect suggests that Osimertinib’s superior CNS penetration may alter the natural history of LM in this population, and its use could be prioritized in high-risk groups. Given the significant reduction of LM incidence with Osimertinib, it is plausible that optimizing TKI selection based on both molecular profile and clinical risk factors could further mitigate the burden of CNS progression in EGFR-mutant NSCLC.

In summary, patients with EGFR mutant NSCLC have a relatively high risk of developing leptomeningeal metastases, especially those with invasive disease characteristics, young age, poor physical condition, and a history of brain metastases. Molecular subtypes and treatment regimens, particularly the use of TKIs that can penetrate the central nervous system, further affect risk. Early identification of high-risk individuals and targeted intervention strategies are crucial for improving clinical outcomes in this challenging patient population.

### 3.2 Clinical presentation and diagnosis of leptomeningeal metastasis

LM represents a devastating complication in patients with EGFR-mutant NSCLC, often manifesting with a wide spectrum of neurological symptoms. The clinical presentation is highly variable, encompassing both typical and atypical neurological features. Common symptoms include persistent headache, nausea, vomiting, altered mental status, cranial nerve palsies, and gait disturbances, reflecting the diffuse infiltration of the leptomeninges by malignant cells (41). In some cases, patients may present with rapidly progressive neuro-ophthalmologic symptoms, such as visual disturbances or diplopia, or with seizures and focal neurological deficits, which can mimic other neurological or inflammatory conditions and thus delay diagnosis (41). Atypical manifestations, such as isolated cognitive impairment, psychiatric symptoms, or even cauda equina syndrome, have also been reported, further complicating the clinical assessment (42). Notably, the presence of LM should be considered in any patient with advanced lung cancer who develops new or unexplained neurological symptoms, regardless of their specificity, as the clinical spectrum is broad and often overlaps with other metastatic or paraneoplastic processes (12). This clinical ambiguity underscores the necessity for a high index of suspicion and a multidisciplinary approach to diagnosis.

Imaging plays a central role in the diagnostic workup for LM, with gadolinium-enhanced magnetic resonance imaging (MRI) being the modality of choice. Classic MRI findings include diffuse or nodular leptomeningeal enhancement along the cerebral sulci, cerebellar folia, and cranial nerves (43). However, imaging findings can be atypical, such as solitary or focal leptomeningeal lesions, gyriform or mass-like enhancements, or even normal scans in the early stages of disease (44). In rare cases, tumor masses may be limited to the arachnoid membrane, as demonstrated in case reports. This suggests that even if magnetic resonance imaging (MRI) results are not diffuse or typical, the imaging findings should be carefully interpreted and the presence of lymphoma should be considered (43). Given that the sensitivity of MRI fluctuates and false negative results are common, clinical doctors cannot rely solely on imaging examinations; A clear diagnosis must combine imaging findings with broader clinical manifestations to avoid overlooking mild or atypical cases (45).

While CSF cytology remains the gold standard, its initial sensitivity is approximately 50%, often limited by low tumor cell shedding and strict pre-analytic constraints (requiring ≥5–10 mL volume and immediate processing) (45). However, the sensitivity of a single CSF cytology is limited, with initial positive rates often below 50%, even in patients with suggestive clinical and radiologic findings (45). Repeated lumbar punctures can improve the diagnostic yield, but logistical and patient-related constraints may limit their feasibility. Ancillary CSF studies, including measurement of tumor markers (such as carcinoembryonic antigen), cytochemical analysis, and flow cytometry, may provide supportive evidence but are not definitive (46). The low sensitivity and potential for false negatives in cytology highlight the need for adjunctive diagnostic approaches, especially in cases where clinical suspicion remains high despite negative cytology.

Molecular and genetic analyses using CSF have emerged as powerful tools to enhance the diagnostic accuracy of LM. Next-generation sequencing (NGS) of CSF-derived cell-free DNA (cfDNA) or ctDNA offers high sensitivity for detecting tumor-specific mutations, such as EGFR mutations, and can reveal the molecular landscape of LM (15, 47). Studies have consistently demonstrated that CSF ctDNA analysis has a higher detection rate of driver and resistance mutations compared to plasma, providing critical information for both diagnosis and therapeutic decision-making (15, 48). Moreover, CSF ctDNA can sometimes detect LM-related genetic alterations earlier than conventional cytology or imaging, suggesting its potential utility in early diagnosis and real-time disease monitoring (49). The integration of CSF molecular profiling into routine clinical practice could, therefore, improve diagnostic sensitivity and enable personalized treatment strategies, although the availability and standardization of these assays remain areas for further development. Implementation Considerations: CSF NGS requires ≥5–10 mL volume, immediate processing; limitations include assay variability and false negatives in low-shedding tumors. Repeat sampling recommended at diagnosis/progression; interpret CSF-plasma discordance as compartmental evolution (expert opinion). If CSF unavailable, rely on plasma NGS cautiously [Grade C; (15, 47)].

Differential diagnosis is crucial, as other malignancies and conditions can mimic LM clinically and radiologically. Sarcomas, for instance, may cause intracranial metastases with leptomeningeal enhancement, mimicking NSCLC spread (50). Other cancers like breast carcinoma, melanoma, and hematologic malignancies. Benign mimics include infections (e.g., aspergillosis, cryptococcosis). These overlaps underscore the need for early, multimodal diagnostics (e.g., repeated CSF NGS) to differentiate and initiate targeted therapy promptly (51).

The diagnosis of LM in EGFR mutant NSCLC requires a combination of clinical symptoms, MRI, and cerebrospinal fluid cytology examination, but each method has its limitations. So, to overcome these limitations, cerebrospinal fluid molecular detection, especially ctDNA analysis based on NGS, has higher sensitivity in detecting meningeal metastasis and potential mutations. Nowadays, timely and accurate diagnosis fundamentally relies on integrating these clinical, imaging, and molecular data to effectively guide patient treatment management.

### 3.3 Prognostic factors of leptomeningeal metastasis

LM in EGFR-mutated NSCLC represents a particularly challenging clinical scenario, with prognosis influenced by a constellation of patient-, disease-, and treatment-related factors. Among the most consistently identified prognostic indicators is the patient’s performance status, often measured by the Karnofsky Performance Status (KPS) or the Eastern Cooperative Oncology Group (ECOG) score. Multiple studies have demonstrated that a higher KPS or lower ECOG score at the time of LM diagnosis is associated with improved overall survival (OS) (52–54). For instance, functional status heavily dictates both survival and treatment viability, with an ECOG PS ≥ 2 or KPS < 70 directly correlating with poorer outcomes. However, severe baseline debilitation should not automatically rule out aggressive management; evidence indicates that interventions like intrathecal chemotherapy can provide substantial benefit to patients with poor performance status (54). Since LM symptoms can evolve unpredictably, clinicians must routinely and objectively evaluate a patient’s performance status to accurately stratify risk and guide ongoing therapy.

The burden of intracranial and extracranial disease at the time of LM diagnosis also plays a pivotal role in determining prognosis. Patients with a higher number of brain metastases, concurrent parenchymal brain involvement, or extensive extracranial organ metastases tend to have significantly shorter survival (52, 55, 56). For example, those presenting with more than three extracranial organ metastases or with synchronous brain parenchymal involvement have been shown to have inferior OS compared to those with limited disease spread (52). The chronological pattern of LM in relation to BM is also relevant: patients developing LM after BM or concurrently with BM generally fare worse than those with isolated or primary LM (55, 57). These findings suggest that the extent and timing of CNS and systemic disease dissemination are integral to risk stratification, and that early, aggressive multimodal interventions may be warranted in patients with limited disease burden.

Molecular typing has become a key component of prognostic assessment for NSCLC-LM. The presence and type of EGFR mutations, as well as concurrent genetic changes, can affect treatment response and survival outcomes. Compared with the L858R mutation in exon 21, classic EGFR mutations such as exon 19 deletion are typically associated with better survival rates (55), while concurrent mutations in TP53, CDKN2A, or MET amplification are linked to poorer prognosis (47, 53). In particular, the detection of these alterations in CSF via next-generation sequencing (NGS) provides a more comprehensive picture of the molecular landscape and may reveal resistance mechanisms not captured in plasma, further refining prognostic assessment (19, 47). Moreover, high levels of genomic instability in CSF cell-free DNA have been associated with shorter OS, suggesting that dynamic liquid biopsy-based monitoring could become an important adjunct to traditional clinical and radiological parameters (51). Combining molecular and clinical data improves prognostic accuracy and directly informs individualized therapy.

Numerous prognostic scoring systems and nomograms have been developed to aid in risk stratification and treatment planning for LM patients. The EANO-ESMO guidelines recommend classification based on CSF cytology and MRI findings, with positive cytology and nodular lesions on contrast-enhanced MRI indicating worse prognosis (58). Additionally, nomograms incorporating variables such as performance status, presence of distant lymph node metastasis, synchronous diagnosis of lung cancer and LM, and systemic immunological markers (e.g., neutrophil-to-lymphocyte ratio) have demonstrated moderate predictive power (C-index ~0.71) (59). However, these models often lack external validation, may not fully account for the molecular heterogeneity of LM, and are limited by the retrospective nature of most supporting studies. As such, while they provide useful frameworks, their application in clinical practice should be individualized and interpreted in the context of evolving molecular and therapeutic landscapes.

In summary, the prognosis of EGFR-mutated NSCLC patients with LM is shaped by a complex interplay of clinical, radiological, and molecular factors. High performance status limited intracranial and extracranial disease burden, favorable EGFR mutation subtypes, and absence of adverse co-mutations are associated with improved survival, while prognostic models offer structured, albeit imperfect, guidance for risk stratification. Ongoing integration of molecular diagnostics and real-world clinical data will likely refine these prognostic tools and enhance personalized management approaches for this high-risk population.

## 4 Impact of TKI resistance mechanisms and staging on treatment decisions for meningeal metastases

### 4.1 Molecular mechanisms of TKI resistance

The molecular mechanisms underlying resistance to EGFR-TKIs in NSCLC are multifaceted and complex, encompassing both EGFR-dependent and EGFR-independent pathways. Among the most well-characterized mechanisms is the emergence of secondary EGFR mutations, notably the T790M mutation, which accounts for approximately 50% of acquired resistance cases following first-generation EGFR-TKI therapy (60). The T790M mutation alters the ATP binding pocket of the epidermal growth factor receptor (EGFR), resulting in a decrease in the efficacy of early generation tyrosine kinase inhibitors (TKIs), making third-generation drugs such as Osimertinib the standard treatment option (60). However, tumors frequently adapt to Osimertinib by acquiring a C797S mutation, which physically blocks the drug’s covalent binding to EGFR (61). This predictable acquired resistance sequence precisely illustrates why continuous molecular monitoring is crucial for adjusting treatment plans as the disease progresses.

In addition to secondary EGFR mutations, activation of the bypass signaling pathway often leads to drug resistance. For example, in some patients who have progressed with third-generation TKI therapy, the incidence of MET amplification is 15 to 20%, which maintains tumor growth through the PI3K/AKT and MAPK pathways independent of EGFR activation downstream (62, 63). Similarly, the strong adaptability of carcinogenic networks, primary or acquired resistance, often involves HER2 gene amplification and PIK3CA gene mutations (64, 65). To improve this situation, many clinical trials are actively testing dual target strategies, such as EGFR-MET bispecific antibodies and PI3K/AKT/mTOR inhibitors, to block these alternative pathways (65, 66). Patients often have multiple resistance mechanisms simultaneously, and treatment must be actively adjusted based on their specific molecular characteristics.

Histological transformation represents another notable resistance mechanism, wherein EGFR-mutant adenocarcinomas undergo lineage plasticity and convert to small-cell lung cancer (SCLC) or, less frequently, to squamous cell carcinoma (SCC) (67, 68). This phenotypic switch frequently coincides with the loss of tumor suppressor genes like RB1 and TP53. Clinically, it completely alters the tumor’s therapeutic sensitivity; EGFR-TKIs become ineffective, forcing a shift to SCLC- or SCC-directed chemotherapy (69). Because repeat biopsies are difficult to obtain upon progression, the actual rate of this transformation is likely underreported. However, molecular profiling of circulating tumor DNA (ctDNA) could offer a less invasive way to catch these shifts early and adjust treatment accordingly.

Beyond established genetic drivers, EGFR-TKI resistance is increasingly tied to epigenetic shifts, noncoding RNAs, and the tumor microenvironment. For instance, the epithelial-to-mesenchymal transition (EMT) drives both intrinsic and acquired resistance by altering gene expression to promote cellular plasticity and drug tolerance (70, 71). Similarly, the tumor immune microenvironment (TIME) actively remodels under TKI pressure, recruiting immunosuppressive cells and utilizing cytokine signaling to evade immune detection (70). Recognizing that tumor-stroma interactions are central to acquired resistance opens the door to novel strategies that target these non-genetic factors alongside standard TKIs.

The diversity of resistance mechanisms has direct implications for subsequent treatment selection. For instance, the detection of T790M mutation after first- or second-generation EGFR-TKI failure supports the use of third-generation inhibitors, whereas identification of C797S or MET amplification may prompt enrollment in clinical trials of fourth-generation TKIs or combination regimens targeting both EGFR and bypass pathways (62, 72). Histological transformation necessitates a shift to conventional chemotherapy, and the presence of EMT or immune escape features may justify the consideration of anti-angiogenic agents or immunotherapy-based combinations, although the latter have shown limited efficacy in EGFR-mutant NSCLC (70, 73). As molecular diagnostic technologies advance, integrating comprehensive genomic and phenotypic profiling into routine clinical practice will be essential for the rational selection of post-resistance therapies and for the development of novel agents capable of overcoming the multifactorial nature of EGFR-TKI resistance.

EGFR-TKI resistance in NSCLC stems from a diverse array of mechanisms, including secondary mutations (T790M, C797S), gene amplifications (MET, HER2), histological transformation, and microenvironmental changes (Table 1). Characterizing these specific drivers is strictly required to guide ongoing clinical management and inform the development of novel combination therapies. Ultimately, overcoming this resistance relies entirely on adapting personalized regimens to each patient’s unique molecular profile.

**Table 1:** Key resistance mechanisms in TKI-Resistant EGFR-Mutant NSCLC with LM.

Mechanism type	Description	Immunological implications	Examples/references
EGFR-dependent mutations	Secondary mutations altering TKI binding sites.	Limited direct immune impact but hypothesized to remodel TIME via signaling changes (60, 61, 70)	T790M, C797S (60, 61)
Bypass pathways	Activation of alternative signaling (e.g., MET/HER2 amplification, PIK3CA mutations).	Promotes immune evasion through immunosuppressive cytokine release and reduced antigen presentation (6, 62–65)	MET amplification, HER2 upregulation (62–65)
Histological transformation	Shift to SCLC/SCC, losing EGFR dependence.	Alters TIME with increased immunosuppressive cells; poor ICI response (67–69, 72)	SCLC transformation with RB1/TP53 loss (67–69)
Tumor microenvironment alterations	EMT, TAMs, exosomal RNAs fostering resistance.	Enhances immune-cold phenotype: low PD-L1, suppressed T-cell infiltration; hypothesized targets for CTLA4/ICI combos (6, 30, 70, 71).	EMT-induced plasticity, TAM-mediated suppression (6, 70, 71).
CNS-specific factors	BBB restriction, CSF compartment evolution.	Creates immune sanctuary; emerging (hypothesized) CTLA4 blockade to boost CNS immunogenicity (30, 31, 89)	Immune evasion in LM (30, 31, 89)

Immunological implications are supplemental and emerging; limited LM-specific data in EGFR-mutant disease [Grade D; (30, 31)]. TKI: Tyrosine Kinase Inhibitor; EGFR, Epidermal Growth Factor Receptor; NSCLC, Non-Small Cell Lung Cancer; LM, Leptomeningeal Metastasis; TIME, Tumor Immune Microenvironment; SCLC, Small Cell Lung Cancer; SCC, Squamous Cell Carcinoma; ICI, Immune Checkpoint Inhibitor; EMT, Epithelial-to-Mesenchymal Transition; TAMs, Tumor-Associated Macrophages; PD-L1, Programmed Death-Ligand 1; CTLA4, Cytotoxic T-Lymphocyte-Associated Protein 4; BBB, Blood–Brain Barrier; CSF, Cerebrospinal Fluid; MET, Mesenchymal-Epithelial Transition factor; HER2, Human Epidermal Growth Factor Receptor 2; PIK3CA, Phosphatidylinositol-4,5-Bisphosphate 3-Kinase Catalytic Subunit Alpha; RB1, Retinoblastoma 1; TP53, Tumor Protein p53.

**Table 2:** Evidence matrix for key therapeutic interventions in LM Post-TKI resistance in EGFR-Mutant NSCLC.

Intervention strategy	Key studies (Refs)	Study design	Key outcomes (mOS/mPFS; other metrics)	Evidence grade*
High-Dose Third-Generation TKIs (e.g., Osimertinib, Furmonertinib)	(17, 18, 22–24, 80, 97)	Prospective Phase II/Retrospective Cohorts/Pivotal Phase III (NCCN Category 1)	mOS ~ 12–15 mo; mPFS ~8 mo; CNS ORR ~ 54–65%	A/B
Combination Strategies (e.g., Osimertinib + Chemo, Amivantamab-based)	(23, 75, 80, 81)	Phase II/Phase III (NCCN Category 1)	mOS ~ 12.6 mo; Intracranial PFS ~ 9.3 mo; ORR 50	A/B
Intrathecal Pemetrexed (Alone or Combined)	(54, 85–87)	Retrospective Cohorts/Case Series	mOS ~ 9–12 mo; Symptom improvement ~64–82%; Neurological PFS ~ 5–10 mo	C/D
Osimertinib + Bevacizumab	(75)	Phase II Prospective	mOS ~ 12.6 mo; Intracranial PFS ~ 9.3 mo; ORR 50%	B
Whole-Brain Radiotherapy (WBRT)	(91)	Retrospective Cohorts	mOS ~ 8–12.6 mo in EGFR+; No added benefit vs. targeted therapy alone; Neurotoxicity risk	C
Proton Craniospinal Irradiation (CSI)	(78)	Phase II RCT	Improved CNS PFS (~8.2 mo vs. 2.3 mo with photon/IFRT); Reduced toxicity	B
Ventriculoperitoneal (VP) Shunting	(94)	Retrospective cohorts	Symptom relief (hydrocephalus) in majority (~90–100% rapid PS improvement post-shunt); mOS extension ~3–6 mo in selected EGFR-mutant cases (enabling continued TKI)	C
Immune Checkpoint Inhibitors (e.g., CTLA4 Blockade)	(30, 31, 73)	Preclinical/Case Reports/Early-Phase	Experimental; Potential for CNS immunogenicity; High toxicity (e.g., neuroinflammation); mOS data limited	D
Staging/Prognostic Guidelines (EANO-ESMO)	(74)	Guideline/Consensus	Stratifies LM by type (nodular/linear); Guides multimodal decisions; mOS 3–11 mo based on PS	A

Evidence Grades: A: Guideline/Consensus-Supported; B: Prospective Trials/Phase II/RCT; C: Retrospective Cohorts; D: Case Reports/Experimental/Preclinical. Outcomes are medians from cited studies, variability due to patient selection. See main text for details. LM, Leptomeningeal Metastasis; TKI, Tyrosine Kinase Inhibitor; EGFR, Epidermal Growth Factor Receptor; NSCLC, Non-Small Cell Lung Cancer; mOS, median Overall Survival; mPFS, median Progression-Free Survival; CNS, Central Nervous System; ORR, Objective Response Rate; CSI, Craniospinal Irradiation; VP, Ventriculoperitoneal; CTLA4, Cytotoxic T-Lymphocyte-Associated Protein 4; RCT, Randomized Controlled Trial; PS, Performance Status; EANO-ESMO, European Association of Neuro-Oncology - European Society for Medical Oncology.

### 4.2 Treatment considerations based on tumor staging

The clinical management of EGFR-mutated NSCLC with LM after TKI resistance is highly influenced by the extent of tumor burden and the pattern of disease progression. Patients with limited leptomeningeal involvement, confined strictly to the CNS, often exhibit different therapeutic responses and prognoses compared to those with extensive LM involving both intracranial and extracranial sites. To standardize clinical assessment and guide the decision-making roadmap, we utilize the following staging definitions: CNS-limited LM: isolated leptomeningeal involvement (nodular/linear MRI enhancement confined to CNS, CSF-positive cytology, no extracranial progression); Extensive LM: concurrent extracranial burden (>3 organ metastases or parenchymal brain metastases). Synchronous progression: intra- and extracranial progression within 3 months; Asynchronous: CNS progression with stable extracranial disease [Aligned with EANO-ESMO; (74)]. In cases of CNS-limited LM, high-dose third-generation EGFR-TKIs such as Osimertinib or Furmonertinib have demonstrated significant clinical benefit, achieving central nervous system response rates exceeding 50% and extending overall survival in multiple retrospective cohorts (18, 52). Notably, high-dose regimens appear superior to standard doses, with median overall survival reaching up to 15 months in some subgroups (18). This suggests that for patients with isolated CNS progression, aggressive CNS-penetrant TKI therapy may provide optimal disease control and delay the need for more toxic interventions.

On the contrary, extensive lymphoma involving the central nervous system and extracranial diseases requires an active multimodal comprehensive treatment plan. These patients have rapid disease progression and poor prognosis, especially when accompanied by brain parenchymal or multiple extracranial metastases (52, 55). The combination of antiangiogenic drugs such as bevacizumab and tyrosine kinase inhibitors can effectively delay the occurrence of brain metastases and improve survival rates, especially in cases where brain metastases occur simultaneously (55, 75). Integrating local treatment methods such as intrathecal chemotherapy or cranial radiation therapy can enhance overall disease control effectiveness. Especially, the combination of cranial radiation therapy and third-generation TKIs can prolong the survival period without LM and reduce the incidence of meningeal metastasis within 2 years, which is superior to the effect of TKIs alone (26). Therefore, for the management of extensive late stage metastatic lesions, it is necessary to adjust the combination therapy strategy according to the specific metastasis situation of the patient.

Whether intracranial and extracranial progression occur synchronously or asynchronously determines the choice of treatment decision. Because synchronous progression often indicates the presence of widespread systemic drug resistance, in order to address the simultaneous occurrence of central nervous system and systemic treatment failure, it is necessary to immediately change the systemic treatment plan, usually by adding chemotherapy, antiangiogenic drugs, or immunotherapy (76, 77). In contrast, patients with asynchronous progression, where CNS disease advances while extracranial disease remains controlled, may continue to benefit from CNS-focused interventions such as high-dose TKIs, intrathecal chemotherapy, or local radiotherapy, potentially postponing systemic regimen changes (18, 52). This distinction underscores the need for careful assessment of disease kinetics and individualized treatment sequencing.

Emerging evidence also suggests that molecular profiling of CSF can inform the choice of targeted therapies in both limited and extensive LM. CSF next-generation sequencing can detect actionable mutations and resistance mechanisms that may not be apparent in plasma, thereby guiding the selection of subsequent TKIs or combination regimens (47, 48). This method is crucial for isolated central nervous system progression, enabling clinicians to identify local resistance pathways and design targeted salvage therapies. Given the variability of drug permeability and tumor resistance, incorporating molecular diagnosis into standard staging algorithms provides a more accurate tool for risk stratification and clinical management.

In summary, the stage and distribution of LM in EGFR-mutant NSCLC after TKI resistance fundamentally shape therapeutic strategy. Patients with CNS-limited LM may derive substantial benefit from high-dose, CNS-penetrant TKIs, while those with extensive or synchronous extracranial progression are more likely to require combination systemic and local therapies. The integration of molecular diagnostics, particularly CSF-based genotyping, offers an additional layer of precision for individualized treatment planning. As research continues to clarify optimal sequencing and combination approaches, multidisciplinary and stage-adapted interventions remain the cornerstone of care for this challenging patient population. Specifically, treatment options for LM encompass systemic therapies (e.g., high-dose third-generation TKIs like Osimertinib for EGFR-mutant cases, immunotherapy for PD-L1-high tumors), radiation [e.g., WBRT for diffuse disease, SRS for focal lesions, proton craniospinal irradiation to minimize toxicity (78)], and surgery (e.g., ventriculoperitoneal shunting for hydrocephalus relief). In refractory or poor-prognosis cases, palliative care focuses on symptom control (e.g., analgesics, anticonvulsants) and quality of life (58, 78).

### 4.3 Treatment pathways based on resistance patterns

The management of EGFR-mutant NSCLC patients who develop leptomeningeal metastases after TKI resistance requires a nuanced understanding of resistance mechanisms and tailored treatment pathways. In the context of a single resistance mechanism, such as the emergence of the EGFR T790M mutation following first- or second-generation TKI therapy, third-generation TKIs like Osimertinib have demonstrated significant efficacy, including in central nervous system (CNS) metastases, due to their ability to penetrate the blood–brain barrier and target T790M-positive clones (72). Unfortunately, acquired resistance to third-generation TKIs remains a major clinical challenge. It usually emerges through new target mutations (such as C797S) or through the activation of alternative pathways, most notably MET or HER2 amplification, PIK3CA mutations, and phenotypic transformation to small cell carcinoma or squamous cell carcinoma (61, 64). Identifying a solitary resistance mechanism allows clinicians to prioritize highly targeted interventions, such as switching TKIs or adding a MET inhibitor for MET amplification (66). Quickly identifying the main driving factors in these extremely complex resistance patterns remains crucial for effectively arranging the sequence of use of rapidly increasing targeted therapeutic drugs.

In contrast, the presence of multiple concurrent resistance mechanisms—such as compound EGFR mutations (e.g., T790M plus C797S), co-occurring pathway alterations (e.g., PI3K/AKT/mTOR activation), or histological transformation—necessitates a multimodal approach. For example, the coexistence of MET amplification and EGFR C797S mutation may require combination regimens that include both MET inhibitors and fourth-generation EGFR TKIs, or the integration of chemotherapy and immunotherapy in select cases (61, 66). The emergence of histological transformation, such as small cell or squamous cell carcinoma, is particularly challenging; these cases often lose EGFR dependence and become refractory to further EGFR inhibition, thus requiring a shift toward platinum-based chemotherapy regimens and, in some instances, radiotherapy (67, 68). The diversity and frequent coexistence of drug resistance mechanisms highlight the necessity of comprehensive molecular and histopathological re-evaluation during disease progression, as the identification of actionable alterations can inform rational combination therapy approaches and facilitate patient participation in clinical trials exploring novel drugs or treatment regimens. It is conceivable that the dynamic evolution of resistance mechanisms under therapeutic pressure may lead to spatial and temporal heterogeneity, further emphasizing the importance of repeated molecular testing to fully capture resistance scenarios and guide adaptive treatment decisions (79).

The choice between tissue biopsy and CSF liquid biopsy (ctDNA) for resistance mechanism identification is a critical consideration in the setting of leptomeningeal metastases. Tissue biopsy, particularly of extracranial lesions, remains the gold standard for histological assessment and can reveal transformations (e.g., to small cell or squamous cell carcinoma) that are not detectable by ctDNA analysis alone (67, 68). However, obtaining tissue from CNS lesions is often impractical or unsafe. Recent advances in CSF ctDNA analysis have enabled the noninvasive detection of resistance mutations, gene amplifications, and even rare alterations in patients with CNS progression, providing a real-time molecular snapshot of the intracranial disease compartment (79). Drug resistance events in the CNS often cannot be detected through standard plasma ctDNA testing due to tumor zoning evolution. However, ctDNA in CSF has significantly higher sensitivity in revealing these hidden and actionable mutations. By routinely incorporating CSF analysis into the diagnosis of meningeal metastases, clinicians can more quickly identify drug resistance drivers and more accurately select targeted therapies. Ultimately, due to the often inconsistent characteristics of central nervous system and systemic diseases, the combination of tissue and fluid biopsy provides the most comprehensive strategy for developing treatment plans tailored to specific regions (79).

In patients with EGFR mutant NSCLC who develop resistance to TKIs, effective management of LM relies entirely on accurate analysis of potential resistance mechanisms. By routinely integrating tissue and CSF ctDNA analysis, clinical doctors can obtain a comprehensive molecular profile to guide personalized multimodal intervention measures. Ultimately, utilizing these comprehensive diagnostic methods to guide personalized targeted therapy remains the most feasible strategy for improving the prognosis of this high-risk population. For disease progression on Osimertinib (including LM), NCCN guidelines endorse platinum-pemetrexed chemotherapy with continuation of Osimertinib (80), for non-CNS-dominant progression, or Amivantamab-based regimens combined with chemotherapy supported by the (81). In LM-specific settings, these systemic backbones are integrated with high-dose third-generation TKIs, intrathecal pemetrexed, and radiotherapy as multimodal therapy.

## 5 Systemic treatment strategies for meningeal metastases

### 5.1 Application of next-generation TKIs in leptomeningeal metastasis

The management of LM in EGFR-mutated NSCLC has evolved significantly with the advent of third-generation TKI, such as Osimertinib and Furmonertinib. Osimertinib is recognized for its superior CNS penetration, which is a crucial factor for efficacy in LM. Per NCCN Category 1 recommendations, Osimertinib monotherapy (FLAURA), Osimertinib plus platinum-pemetrexed (FLAURA2), or Amivantamab plus Lazertinib (MARIPOSA) represent the current standard first-line options that substantially delay CNS progression; high-dose regimens (Osimertinib 160 mg or Furmonertinib 160/240 mg) are particularly relevant in the LM setting due to enhanced CSF exposure (21–24). Clinical studies and retrospective analyses have demonstrated that Osimertinib achieves higher CSF concentrations compared to earlier-generation TKIs, leading to improved intracranial disease control and extended PFS in patients with LM. For example, a retrospective study involving 70 EGFR-mutated NSCLC patients with LM revealed that those treated with third-generation TKIs (Osimertinib 80 mg daily) had a median PFS of 10.8 months, significantly longer than the 5.3 months observed in patients using first- or second-generation TKIs (P:0.019) (82). This PFS benefit underscores the role of Osimertinib as the preferred agent for CNS involvement, especially in the context of LM.

Furmonertinib is a highly permeable and safe tyrosine kinase inhibitor. In a prospective real-world cohort study of 48 patients with EGFR mutant non-small cell lung cancer and meningeal metastasis, high-dose Furmonertinib (240 mg per day) resulted in a median overall survival of 8.43 months, a clinical remission rate of 75%, and an objective imaging remission rate of 50% (28). It can still maintain efficacy in patients who have previously received treatment with other third-generation tyrosine kinase inhibitors, so changing drugs or increasing doses is still a feasible clinical strategy for central nervous system progression, highlighting the potential of individualized and combination therapy regimens.

The efficacy of new-generation TKIs is also closely tied to the molecular landscape of LM, which can be distinctly profiled through CSF-based liquid biopsy. Multiple studies have demonstrated that CSF ctDNA provides a more comprehensive and sensitive assessment of driver and resistance mutations compared to plasma, especially in the context of CNS progression (19, 53). For instance, CSF-based next-generation sequencing (NGS) detects EGFR mutations, TP53, and copy number variations at higher rates in LM than matched plasma samples, guiding the selection of targeted therapies and informing on resistance mechanisms (19). This molecular profiling is critical, as the emergence of compound or rare EGFR mutations, such as L833V/H835L, can influence the response to specific TKIs. A case report highlighted the successful use of Aumolertinib, another third-generation TKI, in a patient with a rare compound EGFR mutation and brain metastasis, achieving a progression-free survival of 18 months (83). These observations suggest that comprehensive molecular diagnostics, especially CSF-based, are indispensable for personalizing TKI therapy and maximizing CNS efficacy.

Third generation TKIs inevitably develop resistance, often through complex genetic mechanisms, which prompts people to search for effective combination therapies. Secondary oncogenic fusion such as ALK rearrangement is a clear and actionable target. For example, a recent case study detailed a patient carrying EGFR exon 19 deletion who developed CNS progression after developing resistance to Osimertinib, driven by newly acquired EML4-ALK fusion. By using the combination therapy of Osimertinib and Alectinib, the patient achieved a complete and long-lasting response, strongly demonstrating that the dual TKI strategy can overcome complex drug resistance and stabilize CNS diseases (84). A literature review of similar cases reported an objective response rate of 66.7% and good tolerability, especially in cases of central nervous system involvement. Therefore, the combination of targeted drugs for secondary driver gene alterations provides significant and feasible benefits for the previously difficult to treat LM.

Clinical doctors are also actively researching how to combine high-dose or combination use of TKIs with adjuvant therapy (such as antiangiogenic drugs) or intrathecal chemotherapy. For example, a large cohort study identified two independent favorable survival predictors in NSCLC-LM after third-generation TKI resistance: combination therapy of Furmonertinib and bevacizumab, and high-dose intrathecal injection of pemetrexed (19). Therefore, a multimodal strategy combining molecular targeted therapy and cytotoxic therapy is needed to maximize control of central nervous system diseases and prolong survival. Looking ahead, these tailored interventions based on combination therapy will undoubtedly become the cornerstone of lymphoma management.

Third generation TKIs such as Osimertinib and Furmonertinib currently dominate the treatment of LM in NSCLC with EGFR mutations, thanks to their strong CNS permeability. However, to effectively address drug resistance, routine molecular testing of CSF is required to accurately guide treatment. Ultimately, combining these TKIs with adjuvant targeted or cytotoxic drugs remains the most critical strategy for overcoming acquired resistance and prolonging survival.

### 5.2 Intrathecal drug therapy

Intrathecal drug administration represents a critical intervention for patients with LM from EGFR-mutant NSCLC who have developed resistance to TKIs. The primary rationale for intrathecal therapy is the limited penetration of many systemic agents across the blood–brain barrier, which restricts their efficacy in the central nervous system. Traditional intrathecal chemotherapeutic agents include methotrexate, cytarabine, and more recently, pemetrexed. Methotrexate and cytarabine have been utilized for decades, with evidence supporting their ability to achieve therapeutic concentrations in the CSF and exert cytotoxic effects on malignant cells. However, their use is associated with significant adverse effects, such as chemical arachnoiditis, myelosuppression, and neurotoxicity, necessitating careful patient selection and monitoring. Prerequisites for intrathecal therapy include assessment of CSF flow (via radioisotope study to ensure no blockages), evaluation of hydrocephalus or elevated ICP (contraindicating if present), and infection screening (e.g., CSF culture). An Ommaya reservoir is preferred over repeated lumbar puncture for frequent dosing to minimize infection risk and patient discomfort. Eligibility typically requires ECOG PS ≤ 2 and no active CNS infection [Grade C; (85, 86)]. Pemetrexed, a multitargeted antifolate, has emerged as a promising alternative due to a more favorable toxicity profile and efficacy in LM from lung cancer. In a retrospective analysis of patients with refractory LM from NSCLC, intrathecal pemetrexed-based multimodal therapy resulted in a median PFS of 9.6 months, with a clinical response observed in approximately 35% of patients and a manageable adverse event profile (85). The integration of intrathecal chemotherapy into a multimodal regimen—including systemic TKIs, chemotherapy, and antiangiogenic agents—has been shown to improve neurological symptom control and prolong survival, especially in patients with EGFR mutations who have exhausted standard options.

The advent of intrathecal administration of targeted agents, particularly third-generation EGFR-TKIs such as Osimertinib, has further expanded the therapeutic landscape for LM. Osimertinib is distinguished by its high central nervous system penetration and potent activity against T790M resistance mutations. Clinical evidence from case reports and small series indicates that intrathecal administration of Osimertinib, in combination with systemic therapy, can lead to rapid alleviation of neurological symptoms and durable disease control in patients with LM following resistance to first- and second-generation TKIs (87). Notably, in a case of EGFR-mutant NSCLC with LM progression after standard-dose Osimertinib, escalating the dose and combining it with intrathecal pemetrexed resulted in sustained neurological improvement and prolonged survival, with toxicity remaining manageable (87). These findings suggest that optimizing the dosing strategy and combining intrathecal with systemic targeted therapies may overcome some resistance mechanisms and improve clinical outcomes.

The clinical application of intrathecal targeted therapy is still in development, with most data coming from case reports and small retrospective series rather than large randomized trials. Nevertheless, an increasing body of evidence supports the feasibility and potential benefits of this approach in highly selected patients, especially those with actionable mutations and preserved performance status. For instance, in a report on rare EGFR-SEPT14 fusion-positive NSCLC and LM patients, the combination of Osimertinib and intrathecal pemetrexed injection led to complete resolution of neurological symptoms, stable imaging results, long-term survival, and no significant side effects (88). The tolerability profile of intrathecal targeted agents appears favorable compared to traditional chemotherapeutics, with fewer reports of neurotoxicity or systemic adverse events. Combining these agents with high-dose systemic third-generation TKIs may further enhance efficacy, as high-dose regimens have been associated with improved response rates and overall survival in LM patients (18). Given the heterogeneity of resistance mechanisms and the dynamic molecular landscape of LM, individualized treatment strategies guided by CSF genomic profiling are likely to maximize therapeutic benefit.

Despite limited prospective data, integrating intrathecal chemotherapy with targeted therapy is increasingly essential for managing LM in NSCLC. Combining systemic TKIs with intrathecal pemetrexed or methotrexate delivers significant clinical efficacy and a controlled safety profile (85, 87). As diagnostic and drug delivery technologies advance, the clinical application of these localized agents will inevitably expand into standardized protocols. Ultimately, molecularly tailored intrathecal therapy remains a crucial, multidisciplinary tool for overcoming TKI-resistant LM.

Intrathecal chemotherapy and targeted agents provide a critical therapeutic lifeline for TKI-resistant, EGFR-mutant NSCLC with LM. Current evidence strongly backs integrating these localized therapies into individualized, multimodal regimens to maximize clinical benefit. Future efforts must focus intensely on optimizing dosing, minimizing toxicity, and refining patient selection to improve survival in this high-risk population.

### 5.3 Immunotherapy and combination treatment strategies

The application of immune checkpoint inhibitors (ICIs) in EGFR-mutant NSCLC with LM has been an area of growing interest, yet significant challenges remain. While ICIs such as PD-1/PD-L1 inhibitors have shown efficacy in various NSCLC subtypes, their benefit in EGFR-mutant cases—particularly those with CNS involvement—is limited. This is largely attributed to the immune-cold microenvironment commonly observed in EGFR-mutant tumors, characterized by low tumor mutational burden and reduced PD-L1 expression, which diminishes the responsiveness to immunotherapy. Furthermore, the BBB and the unique immunological milieu of the CNS further restrict the infiltration and activity of immune effector cells, complicating the therapeutic impact of ICIs in patients with leptomeningeal or brain metastases (89). To overcome these biological barriers and the subsequent failure of standard ICIs, future strategies must utilize novel combinations capable of actively remodeling the CNS tumor immune microenvironment.

In recent years, the feasibility of combining EGFR-TKIs with immunotherapy or chemotherapy has garnered increasing attention to address resistance and improve outcomes in EGFR-mutant NSCLC with CNS involvement. Preclinical and early clinical data suggest that EGFR-TKIs, particularly third-generation agents such as Osimertinib and Aumolertinib, possess favorable CNS penetration and can achieve disease control in patients with brain or leptomeningeal metastases, even after the emergence of resistance mutations like T790M (89, 90). The rationale for combining TKIs with immunotherapy is supported by the hypothesis that targeted therapy may enhance tumor antigen release and immune recognition, thereby sensitizing tumors to ICIs. However, strategies including ICIs (e.g., PD-1/PD-L1 inhibitors) or CTLA4 blockade are currently investigational in this setting due to limited LM-specific data [Grade D]. Known toxicities include neuroinflammation or immune-related encephalitis; furthermore, sequencing cautions strongly recommend avoiding concurrent TKI/ICI administration to prevent severe interstitial lung disease (ILD) risk. Instead, sequential or staggered combination regimens should be explored. Eligibility for these investigational approaches focuses on PD-L1-high tumors and good performance status (ECOG ≤1), with mandatory close monitoring for CNS adverse events [Grade D; (30, 31, 73)]. It is conceivable that sequential or staggered combination regimens, rather than concurrent administration, may improve the safety profile and therapeutic index for these patients.

When acquired resistance and central nervous system progression occur, standard chemotherapy usually becomes the main salvage strategy. Unfortunately, although adding platinum-based doublets to first-generation TKIs can offer selective, moderate benefits, these regimens ultimately fail to provide durable responses, especially for aggressive leptomeningeal metastases (90). Third-generation TKIs, such as Aumolertinib have been shown to induce partial responses and prolong progression-free survival in patients with EGFR-mutated NSCLC carrying complex molecular alterations, including concurrent TP53 mutations and EGFR amplification, even after first-line treatment failure. In a reported case, a patient with EGFR exon 19 deletion, TP53 co-mutation, and leptomeningeal metastasis achieved 12 months of disease control with Aumolertinib after previous TKI and chemotherapy, highlighting the potential of late-generation TKIs to overcome certain resistance mechanisms and providing meaningful clinical benefit in this challenging setting (90). This observation raises the possibility that integrating molecular monitoring of CSF circulating tumor DNA could further refine treatment selection and response assessment for patients with CNS metastases.

The exploration of combination strategies, including the integration of immunotherapy, targeted therapy, and chemotherapy, is ongoing, with the aim of overcoming resistance and improving survival in EGFR-mutant NSCLC with CNS involvement. As fourth-generation TKIs and antibody-drug conjugates (ADCs) transition into standard practice, it remains crucial to effectively integrate them into dynamic molecular-guided regimens. Utilizing these new therapies within a collaborative, multidisciplinary framework offers the most feasible clinical approach to extending the survival of patients with leptomeningeal or brain metastases (89). Given the complexity and heterogeneity of resistance mechanisms, future research should focus on individualized therapeutic approaches that account for tumor genomics, microenvironmental factors, and CNS-specific challenges.

In summary, while immunotherapy alone has limited efficacy in EGFR-mutant NSCLC with CNS metastases, combination strategies involving TKIs, chemotherapy, and novel agents are emerging as promising avenues. Continued research and clinical trials are essential to optimize these regimens, improve patient outcomes, and ultimately enhance the quality of life for this high-risk patient population.

## 6 Local and supportive treatment of meningeal metastases

### 6.1 Role of radiotherapy in leptomeningeal metastasis

Radiotherapy, particularly whole-brain radiotherapy (WBRT) and stereotactic radiosurgery (SRS), has long held a central role in the management of LM from NSCLC, especially in the era before the advent of highly CNS-penetrant targeted therapies. Focal approaches like SRS are considered for limited nodular LM (e.g., few lesions, good PS), WBRT for diffuse linear enhancement or symptomatic disease, and craniospinal irradiation for extensive neuraxis involvement. Integration with systemic therapy involves sequencing RT after TKI to avoid enhanced radiosensitization and toxicity; eligibility includes KPS ≥ 60 and no recent high-dose steroids to reduce edema risk [Grade B/C; (78, 91)]. WBRT is traditionally indicated for patients with diffuse LM, multiple brain metastases, or significant neurological symptoms, as it allows for the treatment of both macroscopic and microscopic disease throughout the entire brain and leptomeninges. However, the efficacy of WBRT in improving overall survival (OS) for patients with EGFR-mutated NSCLC and LM is increasingly questioned. A large retrospective analysis demonstrated that, while WBRT offered a clear survival benefit in wild-type EGFR patients (median OS 8.0 vs. 2.1 months, P: 0.002), no survival benefit was observed in those with EGFR mutations (P: 0.490) (91). This suggests that the molecular profile of the tumor should be a primary consideration when selecting patients for WBRT, and that EGFR-mutated populations may derive limited survival advantage from WBRT alone.

SRS and its variants, such as hypofractionated stereotactic radiotherapy (hfSRT), have emerged as valuable tools for selected patients with focal or nodular LM, particularly when the disease is limited to discrete sites. Recent case reports confirm that hfSRT can drive multiple large leptomeningeal metastases into durable, complete remission. Most notably, this strategy successfully manages highly aggressive histologies—like large cell neuroendocrine carcinoma—without subjecting the patient to the toxicities of WBRT or systemic chemotherapy (92). These findings indicate that, in carefully selected cases with limited LM burden and favorable performance status, SRS/hfSRT may provide durable local control while sparing patients the neurocognitive toxicity associated with WBRT. As radiotherapy techniques continue to evolve, the use of advanced modalities such as proton craniospinal irradiation (pCSI) is gaining traction. Randomized trials have shown that pCSI significantly improves CNS progression-free survival (median 8.2 vs. 2.3 months, *p* < 0.001) and overall survival (median 11.3 vs. 4.9 months, P: 0.04) compared with photon involved-field radiotherapy (IFRT), without an increase in severe toxicity (78). This supports the consideration of pCSI as a preferred option when available, particularly for patients with extensive LM.

The integration and timing of radiotherapy with systemic therapies are increasingly recognized as critical factors in optimizing outcomes for NSCLC patients with LM. Several studies have shown that the combination of targeted therapies, such as third-generation EGFR TKIs, with local radiotherapy can lead to improved disease control and survival. For example, patients receiving concurrent local leptomeningeal therapy (chemotherapy or radiotherapy) and targeted therapy achieved longer progression-free survival (11.4 vs. 6.5 months, P:0.02) and overall survival (18.1 vs. 13.6 months, P:0.04) than those receiving targeted therapy alone (93). Furthermore, the sequencing of radiotherapy and systemic agents may influence efficacy; prior radiotherapy was associated with improved progression-free survival in patients subsequently treated with high-dose Osimertinib for CNS metastases (17). This suggests that radiotherapy may sensitize tumor cells to subsequent systemic therapies or help control local disease progression, potentially enhancing the overall therapeutic effect.

Despite these advances, the role of WBRT in the management of LM, especially in the context of modern targeted therapies, remains controversial. Concerns regarding neurocognitive toxicity and limited survival benefit in certain molecular subgroups have led to a shift toward more individualized approaches. In patients with EGFR-mutated NSCLC and LM, the increasing efficacy of CNS-penetrant TKIs has prompted clinicians to reserve WBRT for those with refractory symptoms, extensive disease not amenable to focal therapy, or rapid neurological decline (77). As a result, the decision to employ WBRT or SRS must be carefully balanced against the patient’s performance status, disease burden, molecular characteristics, and expected quality of life.

Although its use is now highly targeted, radiotherapy remains an essential component of LM management in NSCLC. The synergy between advanced radiation technologies and modern systemic therapies directly facilitates precise, individualized treatment sequencing. Consequently, effectively integrating these modalities requires strict multidisciplinary oversight to maximize survival and neurological preservation.

### 6.2 Intracranial pressure management devices

Elevated intracranial pressure (ICP) is a frequent and life-threatening complication in patients with meningeal metastasis from EGFR-mutant NSCLC following resistance to TKIs. The underlying mechanisms of ICP elevation in this context are multifactorial, involving direct tumor infiltration of the meninges, obstruction of CSF pathways, and impaired CSF absorption, which collectively disrupt normal CSF circulation. Clinically, patients may present with symptoms such as persistent headache, nausea, vomiting, altered mental status, papilledema, and, in severe cases, rapid deterioration of consciousness or coma. The onset of such symptoms often signals advanced disease progression and necessitates prompt intervention to prevent irreversible neurological damage and improve quality of life.

In the management of refractory intracranial hypertension, the use of intracranial decompression devices—most notably ventriculoperitoneal (VP) shunts and intrathecal catheters (such as Ommaya reservoirs)—has become a cornerstone of supportive care. The primary indication for VP shunt placement is obstructive hydrocephalus secondary to leptomeningeal tumor infiltration or blockage of CSF outflow, with the goal of diverting excess CSF from the ventricles to the peritoneal cavity to alleviate pressure (94). Similarly, the Ommaya reservoir, an intrathecal catheter system, is indicated for both decompression and repeated intrathecal administration of chemotherapeutic agents, particularly in patients with diffuse leptomeningeal involvement and recurrent CSF flow obstruction. The decision between these modalities is influenced by the pattern of CSF obstruction, patient performance status, and anticipated need for repeated intrathecal therapy. In practice, the Ommaya reservoir not only facilitates CSF drainage but also allows for direct delivery of agents such as pemetrexed or methotrexate, which has shown efficacy in prolonging progression-free survival in patients with severe meningeal symptoms.

Intracranial decompression devices pose a significant risk of incidence rate and may quickly impair clinical outcomes. To address these challenges, strict adherence to aseptic techniques is required to prevent infections such as ventricular or meningitis, while rapid imaging examinations and surgical interventions are necessary in the event of shunt tube failure. In addition, due to the frequent occurrence of subdural hematoma caused by rapid cerebrospinal fluid drainage, gradual decompression is more favored. When using this hardware device for intrathecal chemotherapy (such as through the Omaye reservoir), clinical doctors must also actively prevent and respond to chemical arachnoiditis and neurotoxicity. Ultimately, the successful combination of decompression devices with targeted intrathecal therapy relied entirely on highly vigilant multidisciplinary collaboration.

Although the literature primarily comprises case reports and retrospective analyses, emerging evidence suggests that early and judicious use of decompression devices in EGFR-mutant NSCLC patients with meningeal metastasis and elevated ICP may not only palliate symptoms but also optimize the delivery and efficacy of systemic and intrathecal therapies. Given the high risk of ICP crises in this population, there is a rationale for considering prophylactic or early intervention in patients with radiological or biochemical evidence of CSF flow compromise before the onset of severe neurological deterioration. This strategy could potentially expand the therapeutic window for multimodal interventions and improve overall outcomes.

In summary, the pathophysiology of intracranial hypertension in meningeal metastasis from EGFR-mutant NSCLC is complex, necessitating a nuanced understanding of decompression device indications, procedural protocols, and complication management. The integration of VP shunts and Ommaya reservoirs into the multidisciplinary care plan, alongside targeted and intrathecal therapies, offers a comprehensive approach to symptom control and potentially improved prognosis in this challenging clinical scenario.

### 6.3 Supportive care and quality of life management

For patients with EGFR-mutant NSCLC who develop LM following TKI resistance, supportive care measures play a critical role in the overall management strategy. The use of glucocorticoids is well-established in alleviating symptoms related to increased intracranial pressure, peritumoral edema, and neurological deficits. In clinical practice, these agents are often administered to rapidly reduce cerebral edema, thereby improving neurological function and quality of life, even though their use should be judicious and tailored to minimize long-term adverse effects. Antiepileptic drugs are indicated in patients who experience seizures, which are not uncommon in the context of LM or brain metastases; their use should be individualized, with careful selection to avoid drug–drug interactions with systemic anticancer therapies. The integration of these symptomatic treatments into the broader care plan ensures that acute complications are managed promptly, which may directly influence a patient’s ability to tolerate and benefit from ongoing oncologic therapies (86).

Although medication can alleviate acute symptoms, sustained functional recovery relies entirely on active neurological rehabilitation therapy. Due to lymphoma and brain metastases often leading to severe motor, cognitive, and speech impairments, these complications greatly reduce patients’ independence. Therefore, it is crucial to have patients receive treatment from physical therapists, occupational therapists, and speech therapists in the early stages of the disease to maximize functional adaptability. In addition, with the continuous extension of patients’ overall survival through new targeted therapies that penetrate the central nervous system, maintaining neurological function is no longer just supportive care—it has become a major clinical goal. Therefore, to truly benefit this growing group of long-term survivors, comprehensive rehabilitation treatment must be directly integrated into standard treatment plans.

For patients facing the dual burden of advanced cancer and impaired neurological function, psychosocial support is another cornerstone of comprehensive management. Diagnosed with LM can cause very serious psychological distress, including anxiety, depression, and existential concerns, and may have a negative impact on treatment adherence and overall health. Obtaining assistance from mental health professionals, receiving structured counseling, and participating in peer support groups are crucial for addressing these psychosocial challenges. In addition, early intervention in palliative care teams has been proven to improve symptom control, facilitate complex decision-making, and provide support to patients and caregivers during the disease progression. With the increasing complexity of oncology and adjuvant therapy, the combination of psychological intervention and palliative care has been regarded as the standard of care for patients with central nervous system involvement, rather than an optional auxiliary means.

In the context of advanced NSCLC with LM, quality of life (QoL) management extends beyond symptom palliation to encompass the preservation of autonomy, dignity, and patient preferences. The use of validated QoL assessment tools allows clinicians to monitor the impact of disease and treatment on physical, emotional, and social domains. Incorporating patient-reported outcomes into routine care facilitates timely identification of unmet needs and guides individualized interventions. Notably, as systemic therapies continue to evolve and extend survival, maintaining QoL emerges as a primary treatment goal, particularly in scenarios where curative options are limited. This approach underscores the paradigm shift towards patient-centered care, where therapeutic decisions are informed not only by efficacy data but also by the lived experiences and priorities of patients and their families.

For drug-resistant epidermal growth factor receptor mutant non-small cell lung cancer with brain metastasis, its complex condition requires a proactive and comprehensive nursing model. In addition to disease modifying drugs, true clinical success lies in combining targeted symptom management with enhanced neurological rehabilitation, psychosocial care, and ongoing quality of life assessment. With the advancement of the medical field and the extension of patient survival, establishing such a strong support framework can ensure that extending survival does not come at the expense of patients’ dignity and daily life functions.

## 7 Multidisciplinary decision-making and clinical technical roadmap

### 7.1 Role of multidisciplinary teams (MDT) in treatment decisions

The management of EGFR-mutated NSCLC patients who develop LM after TKI resistance is exceptionally complex, necessitating a coordinated MDT approach. Typically, the MDT comprises medical oncologists, neurosurgeons, radiation oncologists, neuroradiologists, and molecular pathologists. This collaborative model enables comprehensive assessment and individualized planning, integrating systemic therapy, local interventions, and molecular diagnostics. For instance, medical oncologists play a pivotal role in selecting and sequencing targeted therapies and chemotherapeutic regimens, while neurosurgeons may be involved for diagnostic lumbar punctures or palliative interventions in cases of increased intracranial pressure or hydrocephalus. Radiation oncologists contribute to the management of symptomatic brain or meningeal lesions through modalities such as whole-brain radiotherapy or stereotactic radiosurgery, whereas neuroradiologists provide critical input for imaging interpretation and disease monitoring. The integration of molecular pathologists is increasingly vital, particularly for interpreting next-generation sequencing (NGS) results from CSF and plasma, which guide the selection of subsequent targeted therapies and help elucidate resistance mechanisms (19, 51). When each specialty contributes its expertise, the MDT is better positioned to tailor therapy to the patient’s evolving clinical and molecular profile, potentially improving survival and quality of life.

The MDT’s decision-making process is especially advantageous in the context of complex cases, such as those involving multiple resistance mutations or ambiguous radiographic findings. The workflow typically begins with a comprehensive review of the patient’s clinical status, prior treatment history, imaging studies, and molecular diagnostic results. For example, the use of CSF ctDNA genotyping has proven superior to plasma in identifying actionable mutations and resistance mechanisms in LM, often revealing genetic heterogeneity and alterations that are not detectable in extracranial disease sites (15, 48). This information is critical for the MDT to determine the appropriateness of switching to alternative TKIs (such as moving from Osimertinib to a second-generation agent like Afatinib or Dacomitinib), considering combination regimens (e.g., PARP inhibitors plus TKIs), or integrating local therapies such as intrathecal chemotherapy or radiotherapy (76, 95). The MDT’s ability to synthesize these diverse data points into a unified management plan is particularly important given the rapid clinical deterioration often seen in LM, where delays or suboptimal choices can have profound consequences. It is plausible that the increasing availability of CSF-based genomic profiling will further enhance the MDT’s capacity to personalize therapy, especially as novel resistance mechanisms and therapeutic targets are identified.

The advantages of the MDT approach are further underscored by its impact on prognosis and patient-centered care. Multivariate analyses from large cohorts have identified factors such as performance status, the use of high-dose third-generation EGFR-TKIs, and the addition of agents like bevacizumab as independent predictors of improved survival in LM patients (18, 19). The MDT is uniquely equipped to weigh these prognostic factors alongside patient preferences and comorbidities, facilitating shared decision-making and the timely adaptation of treatment strategies in response to disease progression or treatment-related toxicity. In this process, patient counseling is paramount. Discussions should cover realistic prognosis (median OS 3–11 months), treatment benefits/risks (e.g., neurological improvement vs. toxicity), and goals of care. It is essential to emphasize shared decision-making, incorporating patient values, performance status, and family support to balance aggressive therapy with palliative options (96). The true value of the MDT lies in its capacity for real-time, cross-specialty communication. This ongoing dialogue allows clinicians to actively monitor treatment response, seamlessly manage toxicities, and coordinate supportive care. Because leptomeningeal disease evolves rapidly and continuously acquires new resistance mutations, rigid treatment pathways often fail; instead, the MDT’s ability to iteratively reassess and pivot is paramount. As new therapeutic modalities emerge, grounding them within this collaborative structure will drastically sharpen treatment selection and improve survival.

For NSCLC patients who are TKI resistant and have EGFR mutations, a MDT model is an absolute clinical necessity for managing LM. By integrating interdisciplinary expertise with advanced molecular diagnostic techniques, MDT can drive highly personalized interventions to address the complex biological characteristics of diseases. Therefore, this collaborative framework can maximize the identification of effective salvage therapies and ensure strict and comprehensive care throughout the entire process from diagnosis to disease progression.

### 7.2 Design and application of clinical decision-making roadmap

The design of a clinical decision-making roadmap for EGFR-mutant lung cancer patients with LM after TKI resistance is rooted in a multifactorial assessment that incorporates disease staging, resistance mechanisms, and patient-specific factors. The roadmap must begin with precise staging of the CNS involvement, integrating both radiological findings and CSF cytology, as these directly impact the selection and sequencing of subsequent interventions. Molecular profiling, particularly through next-generation sequencing (NGS) of CSF-derived ctDNA, provides a comprehensive landscape of driver and resistance mutations, often revealing a higher abundance and diversity of actionable alterations compared to plasma or tissue samples (15, 51). This enables clinicians to tailor interventions to the evolving molecular profile of LM, especially in the context of acquired resistance to first-, second-, or third-generation EGFR-TKIs. The integration of such molecular data with clinical parameters—such as performance status (ECOG/KPS), presence of extracranial disease, and neurological symptom burden—serves as the foundation for a personalized treatment algorithm (53).

**Figure 1:**
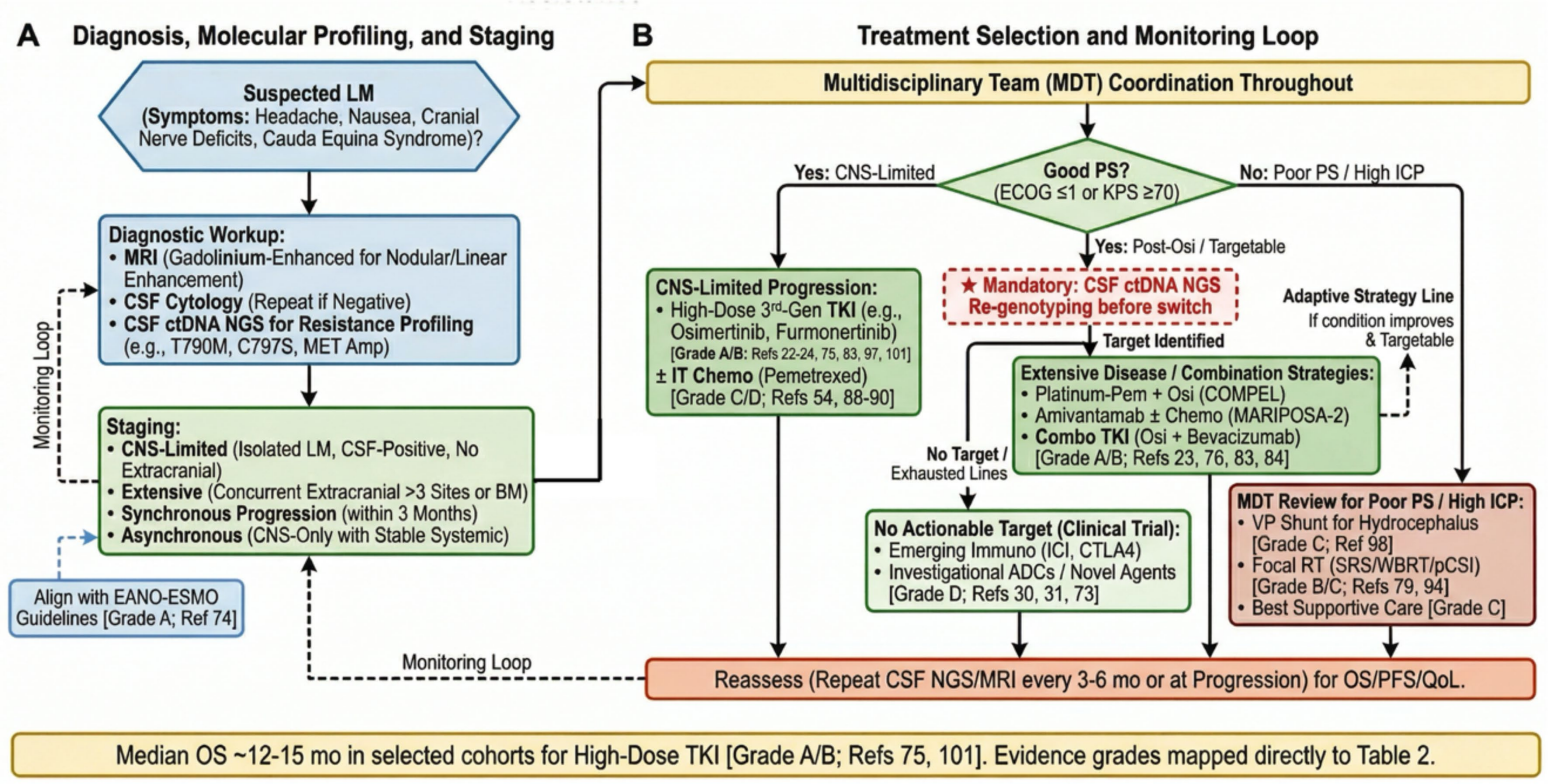
Panel **A** illustrates the diagnostic workup (gadolinium-enhanced MRI, CSF cytology, and mandatory CSF ctDNA NGS) and EANO-ESMO-aligned staging definitions for suspected LM. Panel **B** presents the NCCN-aligned treatment selection algorithm with multidisciplinary team (MDT) coordination. For patients with CNS-limited disease and good performance status (ECOG ≤1 or KPS ≥ 70), high-dose third-generation TKIs are recommended. A dedicated post-Osimertinib progression branch (NCCN Category 1) incorporates combination strategies. The mandatory serial CSF ctDNA NGS re-genotyping before any regimen switch is highlighted. Patients with poor performance status or high intracranial pressure enter supportive/palliative pathways. All patients enter a dynamic monitoring loop with repeat CSF NGS and MRI every 3–6 months or at progression. Evidence grades are mapped directly to Table 2. Median OS is approximately 12–15 months in selected cohorts treated with high-dose TKI therapy. Abbreviations: LM: leptomeningeal metastasis; EGFR: epidermal growth factor receptor; NSCLC: non-small cell lung cancer; TKI: tyrosine kinase inhibitor; MDT: multidisciplinary team; PS: performance status; ECOG: Eastern Cooperative Oncology Group; KPS: Karnofsky Performance Status; CSF: cerebrospinal fluid; ctDNA: circulating tumor DNA; NGS: next-generation sequencing; IT: intrathecal; Osi: Osimertinib; COMPEL: continuation of Osimertinib plus Platinum-Pemetrexed; MARIPOSA-2: Amivantamab plus Chemotherapy after Osimertinib Progression; WBRT: whole-brain radiotherapy; SRS: stereotactic radiosurgery; pCSI: proton craniospinal irradiation; VP: ventriculoperitoneal; ICP: intracranial pressure; OS: overall survival; PFS: progression-free survival; QoL: quality of life; ADCs: antibody-drug conjugates.

The technical roadmap (Figure 1) itself is structured as a stepwise algorithm: (1) confirm LM diagnosis and EGFR mutation status using CSF NGS, (2) assess prior TKI exposure and resistance patterns (e.g., T790M, C797S, bypass pathway activation), (3) evaluate patient performance status and comorbidities, and (4) stratify patients into intervention pathways based on molecular and clinical findings. The roadmap now includes a dedicated post-osimertinib progression branch referencing NCCN Category 1 recommendations (COMPEL and MARIPOSA-2), with mandatory CSF ctDNA NGS re-genotyping before switching to amivantamab, fourth-generation TKIs, or ADCs. For patients with actionable EGFR mutations and good performance status, high-dose third-generation EGFR-TKIs (such as Osimertinib, Aumolertinib, or Furmonertinib) are prioritized, with recent evidence demonstrating superior intracranial response rates and overall survival compared to standard dosing, even after prior TKI resistance (18, 97). In situations where third-generation TKI resistance is confirmed and actionable resistance mechanisms (e.g., EGFR amplification, MET amplification, TP53 co-mutations) are identified in CSF, the roadmap incorporates molecularly matched combination strategies, such as the addition of bevacizumab, intrathecal chemotherapy, or PARP inhibitors in select cases (19, 76). This approach is supported by the observation that patients with good performance status and those receiving multi-modal therapy (e.g., high-dose pemetrexed, targeted combinations) achieve improved survival outcomes (19, 53).

The operationalization of this roadmap in clinical practice requires a multidisciplinary workflow and dynamic reassessment at each decision node. For example, following LM diagnosis and molecular characterization, a patient with EGFR L858R/TP53/ERBB2 mutations and Osimertinib resistance may be considered for a combination of Dacomitinib and olaparib, as illustrated by a case achieving 6 months of progression-free survival and 13 months overall survival post-LM diagnosis (76). Alternatively, in patients with LM progression on Osimertinib and detection of EGFR amplification in CSF, switching to high-dose third-generation TKI or integrating anti-angiogenic agents may be beneficial (19, 97). The roadmap also emphasizes serial monitoring of CSF ctDNA to dynamically track molecular evolution and guide timely therapeutic adjustments, a strategy that has shown strong correlation with clinical response and progression (15, 19). It is increasingly apparent that the use of CSF-based molecular profiling not only enhances detection of actionable mutations but also enables real-time adaptation of treatment plans, potentially mitigating the rapid clinical deterioration seen in LM.

In terms of workflow, the application of roadmap starts with baseline assessment and molecular typing and then stratifies patients into standard treatment group or intensified treatment group based on mutation status and resistance pattern. For patients with poor physical condition or extensive extracranial diseases, supportive care and symptom management are prioritized, while patients with actionable targets and functional preservation are guided to receive active multimodal interventions. The roadmap also includes regular reassessment through imaging, cerebrospinal fluid cytology, and circulating tumor DNA dynamics to detect early progression or the emergence of new resistance mechanisms, to upgrade or adjust treatment plans in a timely manner. It is worth noting that the roadmap is designed iteratively to enable personalized decision-making as new clinical or molecular data emerges.

For patients with epidermal EGFR mutant NSCLC with meningeal metastasis and resistance to TKIs, effective management depends on dynamic, evidence-based algorithms tailored to the individual. By continuously integrating disease staging, molecular resistance mechanisms, and patient specific factors, clinicians can guide precise multimodal interventions. Ultimately, anchoring this personalized approach to continuous molecular diagnostics based on CSF provides the most powerful strategy for overcoming drug resistance and prolonging survival.

### 7.3 Optimization and challenges of individualized treatment strategies

The implementation of individualized therapy for EGFR-mutant NSCLC with leptomeningeal metastasis following TKI resistance faces several tangible barriers, including drug accessibility, patient adherence, and the heterogeneity of resistance mechanisms. Despite the clinical benefits of sequential and combination TKI strategies, real-world data reveal that access to third-generation agents such as Osimertinib and novel combination regimens is often limited by high costs, regulatory hurdles, and regional disparities in drug approval and reimbursement (98). Additionally, patient adherence is challenged by the complexity of treatment regimens, the need for frequent molecular re-assessment, and the burden of adverse effects, especially as combination therapies and multi-line treatments become more common. For instance, even with the advent of third-generation TKIs, the duration of benefit is often curtailed by the emergence of acquired resistance, necessitating careful longitudinal monitoring and dynamic adaptation of therapy—requirements that can strain both healthcare systems and patient compliance (99). These obstacles are compounded by the necessity for repeated biopsies and advanced molecular profiling, which may not be readily available in all clinical settings, thus impeding the full realization of individualized treatment plans.

Optimizing future therapies requires seamlessly pairing novel drugs with exhaustive molecular subtyping. To bypass tertiary mutations like C797S, clinicians anticipate fourth-generation EGFR-TKIs, alongside emerging bispecific antibodies like Amivantamab and ADCs like Patritumab deruxtecan (100, 101). Crucially, deploying these agents effectively demands continuous genomic profiling. Because compound EGFR mutations and concurrent MET or PIK3CA alterations heavily drive resistance, liquid biopsies remain essential for tracking real-time tumor heterogeneity (102). Integrating these advanced sequencing tools into routine care will directly translate trial-level precision into tangible survival benefits for treatment-refractory patients.

Another critical aspect of future optimization is the integration of real-world data (RWD) to inform clinical decision-making and guideline development. Large-scale retrospective and prospective cohort studies have begun to shed light on the effectiveness and safety of various sequencing and combination strategies outside the controlled environment of clinical trials (103). These data are particularly valuable for understanding the impact of patient comorbidities, performance status and treatment adherence on outcomes, as well as for identifying subgroups that may disproportionately benefit or be at risk from specific interventions. For instance, real-world analyses have shown that a sequential TKI strategy under biomarker-guided transitions can yield better survival outcomes in selected patients, especially when resistance mechanisms such as T790M are promptly identified and targeted (103). As more RWD are collected and integrated with clinical trial findings, the ability to tailor therapy to individual patient characteristics and resistance patterns is likely to improve, supporting the evolution of true precision oncology.

Despite these advances, significant challenges remain. The heterogeneity of resistance mechanisms—spanning EGFR-dependent mutations, bypass pathway activations, and phenotypic transformations—demands a highly adaptable and multidisciplinary approach to care (102). Furthermore, disparities in healthcare infrastructure, access to molecular diagnostics, and the availability of new therapies persist across regions and healthcare systems, limiting the equitable implementation of individualized strategies. Addressing these gaps will require coordinated efforts among clinicians, researchers, policymakers, and industry stakeholders to ensure that innovations in drug development, molecular profiling, and data integration translate into tangible benefits for all patients. As the landscape of EGFR-mutant NSCLC continues to evolve, the convergence of novel therapeutics, precise molecular diagnostics, and robust real-world evidence will be pivotal in overcoming current barriers and realizing the full potential of individualized treatment strategies.

The key to effectively managing drug-resistant EGFR mutant non-small cell lung cancer with brain metastasis lies in overcoming issues such as limited drug access, poor patient compliance, and severe tumor heterogeneity. To promote the development of this field, researchers must prioritize novel targeted drugs, strengthen molecular typing, and actively utilize real-world data. Ultimately, active interdisciplinary collaboration remains the only feasible strategy to overcome these multifaceted challenges and effectively improve patient outcomes.

## 8 Conclusion

In conclusion, the management of LM following TKI resistance in EGFR-mutant NSCLC represents a complex clinical challenge that necessitates a nuanced, individualized approach. From an expert perspective, the evolution of therapeutic strategies in this domain highlights the critical importance of integrating tumor staging, resistance mechanisms, and the patient’s overall systemic condition into personalized treatment plans. This tailored approach ensures that interventions are not only targeted but also considerate of the heterogeneity inherent in both tumor biology and patient health status.

To effectively treat LM, a multi-pronged approach is needed, as a single therapy cannot control this invasive disease. Nowadays, clinical collaborative therapy relies on multimodal strategies, actively integrating intrathecal drug therapy, radiotherapy, and surgical decompression. Although these combination therapies greatly improve patients’ quality of life, their success depends entirely on strict patient selection and precise timing of intervention to strike a balance between clinical benefits and serious adverse reactions.

Furthermore, the role of MDT and the application of clinical decision-making algorithms or roadmaps cannot be overstated. These collaborative frameworks facilitate the synthesis of diverse expertise—from medical oncology, radiation oncology, neurology, to palliative care—ensuring that treatment strategies are both evidence-based and contextually appropriate. This integrative approach promotes standardization of care, reduces variability in clinical practice, and enhances therapeutic efficacy, ultimately translating to improved patient outcomes.

To improve clinical outcomes, researchers must urgently prioritize novel therapies that can penetrate the central nervous system, including next-generation tyrosine kinase inhibitors, immunotherapy, and advanced drug delivery systems. Meanwhile, integrating real-world data is crucial for capturing the true therapeutic effects of different populations. By integrating these real-world evidence into standard practices, clinicians can go beyond controlled trials, accurately refine prognostic models, identify predictive biomarkers, and provide highly personalized interventions.

The clinical treatment of TKI resistant and EGFR mutant NSCLC patient with LM requires a highly personalized and multidisciplinary approach. This transformation abandons vague treatment methods and instead adopts a solid framework based on data, directly translating the latest research results into practical intervention measures. Therefore, breaking through existing treatment bottlenecks to improve long-term prognosis now relies entirely on active interdisciplinary collaboration.

## Glossary

LM: Leptomeningeal Metastasis
TKI: Tyrosine Kinase Inhibitor
EGFR: Epidermal Growth Factor Receptor
NSCLC: Non-Small Cell Lung Cancer
BBB: Blood–Brain Barrier
CNS: Central Nervous System
CSF: Cerebrospinal Fluid
ctDNA: Circulating Tumor DNA
NGS: Next-Generation Sequencing
MDT: Multidisciplinary Team
OS: Overall Survival
PFS: Progression-Free Survival
PS: Performance Status
QoL: Quality of Life
RT: Radiotherapy
TP53: Tumor Protein p53
VP: Ventriculoperitoneal
Chemo: Chemotherapy
ECOG: Eastern Cooperative Oncology Group
Ext: Extensive
Immuno: Immunotherapy
IT: Intrathecal
KPS: Karnofsky Performance Status
Lim: Limited
MET: Mesenchymal-Epithelial Transition factor
MRI: Magnetic Resonance Imaging
TIME: Tumor Immune Microenvironment
SCLC: Small Cell Lung Cancer
SCC: Squamous Cell Carcinoma
ICI: Immune Checkpoint Inhibitor
EMT: Epithelial-to-Mesenchymal Transition
TAMs: Tumor-Associated Macrophages
PD-L1: Programmed Death-Ligand 1
CTLA4: Cytotoxic T-Lymphocyte-Associated Protein 4
HER2: Human Epidermal Growth Factor Receptor 2
PIK3CA: Phosphatidylinositol-4,5-Bisphosphate 3-Kinase Catalytic Subunit Alpha
RB1: Retinoblastoma 1
SRS: Stereotactic Radiosurgery
WBRT: Whole-Brain Radiotherapy
CSI: Craniospinal Irradiation
IFRT: Involved-Field Radiation Therapy
RCT: Randomized Controlled Trial
EANO-ESMO: European Association of Neuro-Oncology - European Society for Medical Oncology
